# Differential gene expression analysis of dasatinib-induced colitis in a patient with chronic myeloid leukemia followed for 3 years: a case report

**DOI:** 10.1186/s12876-020-01584-6

**Published:** 2021-01-06

**Authors:** Naoki Oshima, Yoshiyuki Mishima, Kotaro Shibagaki, Kousaku Kawashima, Norihisa Ishimura, Fumiyoshi Ikejiri, Chie Onishi, Takahiro Okada, Masaya Inoue, Ichiro Moriyama, Junji Suzumiya, Yoshikazu Kinoshita, Shunji Ishihara

**Affiliations:** 1 Department of Gastroenterology, Shimane University School of Medicine, 89-1, Enya-cho, Izumo, Shimane 693-8501 Japan; 2 Division of Gastrointestinal Endoscopy, Shimane University Hospital, 89-1, Enya-cho, Izumo, Shimane 693-8501 Japan; 3 Innovative Cancer Center, Shimane University Hospital, 89-1, Enya-cho, Izumo, Shimane 693-8501 Japan; 4 Department of Medicine, Steel Memorial Hirohata Hospital, 3-1, Yumesaki-cho, Himeji, Hyogo 671-1122 Japan

**Keywords:** Chronic myeloid leukemia, Dasatinib, Tyrosine kinase inhibitor, Colitis, Gene expression analysis, Case report

## Abstract

**Background:**

Dasatinib is a second-generation tyrosine kinase inhibitor (TKI) developed for treatment of patients with chronic myeloid leukemia (CML). The drug has been shown to act as a potent multikinase inhibitor by blocking not only the BCR-ABL1 gene sequence but also the SRC kinase family, though unexpected adverse events such as pleural effusion have recently been reported in patients undergoing treatment with dasatinib. Hemorrhagic colitis is a unique gastrointestinal adverse events associated with dasatinib and its pathogenesis remains poorly understood.

**Case presentation:**

We report here a case of dasatinib-induced asymptomatic colitis in a patient with CML, who showed no exacerbation in careful observations and maintained deep molecular response (DMR) during a 3-year period. In addition, we performed transcriptome analysis of inflamed colonic mucosa specimens to clarify the possible mechanism of colitis that develops in association with dasatinib administration. Our results demonstrated that differential gene expression, especially lymphocyte-associated genes and chemokines, is substantially involved in inflammation of colonic mucosa in affected patients.

**Conclusion:**

Dasatinib induces immune-mediated colitis following lymphocyte infiltration.

## Background

Chronic myeloid leukemia (CML) is a clonal myeloproliferative disorder of hematopoietic stem cells, and characterized by overproduction of myeloid cells as well as the presence of the Philadelphia (Ph) chromosome, which leads to formation of the *BCR-ABL1* oncogene [1]. The chimeric BCR-ABL1 protein produced by this fusion gene is a constitutively active tyrosine kinase enzyme that suppresses apoptosis and induces replication via several downstream pathways, such as RAS, RAF, STAT, and MYC. Following the introduction of imatinib, administration of tyrosine kinase inhibitors (TKIs) targeting BCR-ABL1 has become standard therapy for CML [2]. These drugs prevent progression to an advanced phase in most patients, and often have excellent effects on disease burden and overall survival of CML patients [3].

Dasatinib, a second-generation TKI, strongly inhibits not only BCR-ABL1, but also SRC kinases, c-KIT, EPHA2, and platelet-derived growth factor receptor-β (PDGFR-β), indicating its superior potency for blocking BCR-ABL1 protein, while it has also been shown to induce faster and deeper clinical response as compared with imatinib [4, 5]. On the other hand, commonly observed adverse events include myelosuppression, transaminitis, fluid retention, and gastrointestinal (GI) disorders, such as nausea and diarrhea, though they are generally well tolerated. Rare cases with unique adverse events including lower GI bleeding due to hemorrhagic colitis during dasatinib treatment have also been reported [6–11]. Moreover, a recent study conducted in Japan revealed that 6 (33%) of 18 CML patients who received dasatinib developed hemorrhagic colitis without symptoms [12]. That incidence of dasatinib-induced colitis was higher than expected, suggesting that clarification of the mechanism for colitis caused by dasatinib is crucial. Previous studies have indicated that dasatinib-induced colitis may be associated with immune reactivity to lymphocytes including T cells by inhibiting immune regulatory kinases [7]. However, the pathogenesis remains unclear because evidence was only obtained from immunostaining of colonic mucosal tissue samples obtained during a colonoscopy examination. Once clinical diagnosis of dasatinib-induced colitis is made, the attending physician may also tend to stop treatment with the drug, though most affected patients do not have abdominal symptoms including bloody stool. Therefore, few cases without interruption of dasatinib have been observed over a long term.

We report here dasatinib-induced asymptomatic colitis in a CML patient who safely continued dasatinib treatment over a period of 3 years. In addition, we performed analysis of inflamed colonic mucosa gene expression to elucidate the details of colitis that develops in patients receiving dasatinib.

## Case presentation

### Case presentation

**Fig. 1:**
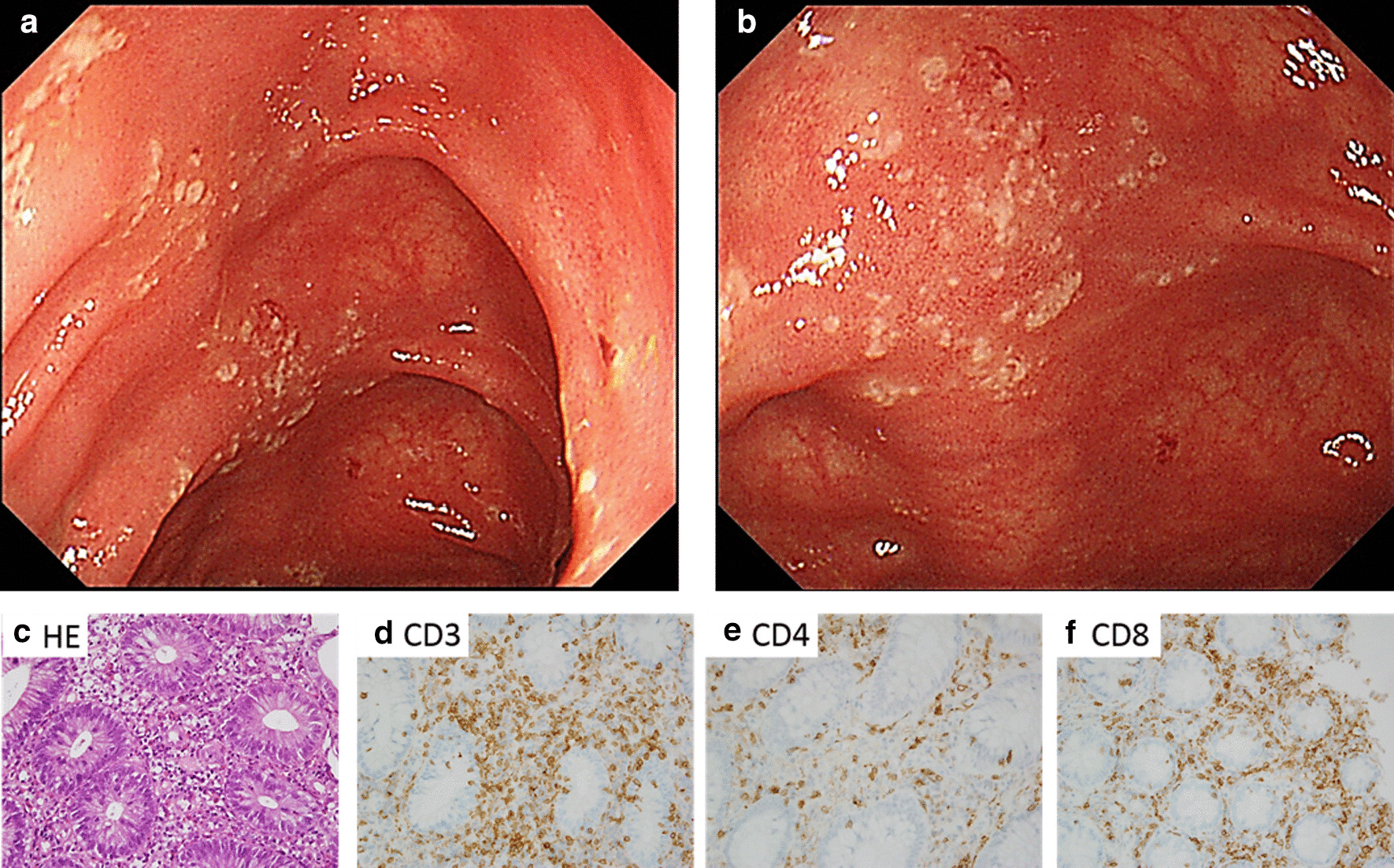
Endoscopic and histological findings showing dasatinib-induced colitis. Colonoscopy revealed multiple shallow areas of erosion with yellow exudate, which appeared to occur in a longitudinal direction in the colon (**a**: normal, **b**: magnified). Hematoxylin and eosin staining of colonic biopsy specimens revealed increased lymphocytic infiltration with cryptitis in the lamina propria (**c**). Immunohistochemistry revealed a high level of staining of CD3 + CD8 + cells, while CD3 + CD4 + cells were only weakly stained (**d**–**f**). HE: hematoxylin and eosin

A previously healthy 56-year-old woman was diagnosed with chronic myeloid leukemia in the accelerated phase (CML-AP) at the age of 53 years. She soon began induction therapy with dasatinib at a daily dose of 100 mg and successfully achieved a deep molecular response (DMR), defined according to the international scale as ≤ 0.01% BCR-ABL1 transcripts, within 12 months. In peripheral blood samples, marked lymphocytosis was observed at 9 months after initiation of dasatinib, which continued throughout the period of therapy. However, other adverse events including myelosuppression were not observed during dasatinib treatment. At 12 months after starting dasatinib, fecal occult blood was detected at a medical check-up for the first time, which the patient had not previously noticed. Furthermore, she reported no gastrointestinal symptoms, such as abdominal pain, diarrhea, or hematochezia, at that examination. Stool cultures were negative for pathogenic enteric bacteria and parasites, and the cytomegalovirus (CMV) antigen C7-HRP was negative in peripheral blood leukocytes. A total colonoscopy was performed and no active bleeding was seen in the colon. However, multiple shallow areas of mucosal erosion with yellow exudate and congested erythematous lesions, which seemed to occur in a longitudinal direction from the transverse to descending colon, were noted (Fig. 1a, b). Mucosal biopsy samples from areas of erosion were obtained and histological results showed increased lymphocytes infiltration to the lamina propria with cryptitis (Fig. 1c). Immunohistochemical (IHC) staining for CD3 and CD8 was strongly positive, while that for CD4 was relatively weak. These observations led to a clinical diagnosis of colitis associated with dasatinib administration. Although patients with drug-induced colitis are typically required to stop the medication, dasatinib was continued without interruption or dose adjustment in the present patient because of the absence of gastrointestinal symptoms, such as bloody stool and abdominal pain, and peripheral blood test findings were nearly within normal ranges, except for leukocytosis.

**Fig. 2:**
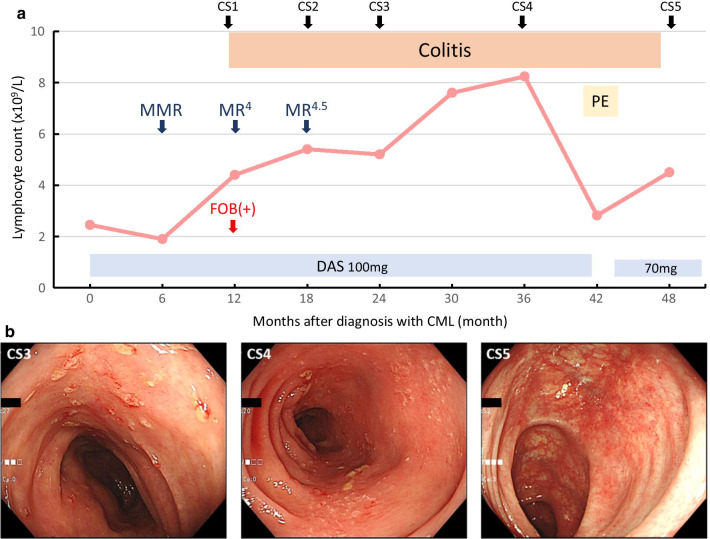
(**a**) Summary of clinical course of the present patient. (**b**) Follow-up colonoscopy images obtained at 12 (CS3), 24 (CS4), and 36 months (CS5) after diagnosis of colitis associated with dasatinib administration. CS, colonoscopy; PE, pleural effusion; DAS, dasatinib; MMR, major molecular response; defined as ≤ 0.1% (MMR), ≤ 0.01% (MR^4^), ≤ 0.0032% (MR^4.5^) BCR-ABL1 transcripts according to the international scale

Endoscopic follow-up examinations were performed at 6, 12, and 24 months after the diagnosis of colitis, each of which showed no exacerbation of inflammation in the colon (Fig. 2). During the follow-up period, the patient had no abdominal symptoms, while marked lymphocytosis was continuously observed. At 30 months after the diagnosis of colitis, a small amount of left pleural effusion without respiratory symptoms was noted, along with dyspnea and a cough during a routine chest X-ray examination. Since dasatinib is thought to cause pleural effusion, the drug was temporarily discontinued. Pleural effusion immediately disappeared along with improvement of lymphocytosis, thus a lower dose of dasatinib (70 mg daily) was started, which maintained the DMR. At 36 months after the initial diagnosis of colitis, follow-up endoscopy revealed that the shallow erosion areas were mostly improved and colonic mucosa was in a near-normal state. Biopsy samples were obtained from colonic mucosa and histological results showed that lymphocytic infiltration was significantly lower in the lamina propria as compared to the past findings of inflamed mucosa of the colon.

### Microarray analysis of expression profiles in colonic biopsy specimens

Colonic mucosal tissue samples were obtained from areas of shallow erosion during diagnostic and follow-up colonoscopy examinations. Following the biopsy, the specimens were placed in RNAlater solution (Qiagen, Valencia, USA) at room temperature and then stored at -80 °C until gene expression profiling. Microarray analysis (Affymetrix, Inc., Santa Clara, USA) was performed according to the manufacturer's instructions. Briefly, total RNA was extracted using an RNeasy Micro kit (Qiagen) and then evaluated with an Agilent 2100 Bioanalyzer (Agilent Technologies, Santa Clara, USA). Gene expression profiles were assessed using microarray technology with a Human Gene 2.0 ST Array (Affymetrix, Inc.). Data analysis was performed using Microarray Data Analysis Tool, ver. 3.2 (Filagen, Nagoya, Japan). To normalize variations in staining intensity between the microarrays, the average difference for all genes with a given microarray was divided by the median of all measurements with that microarray. In addition, to identify more specific biological processes, we performed gene ontology (GO) enrichment analysis of differentially expressed genes in colonic inflamed mucosa. For comparing differences between samples, statistical analysis was performed using a two-tailed Fisher’s exact test.

**Fig. 3:**
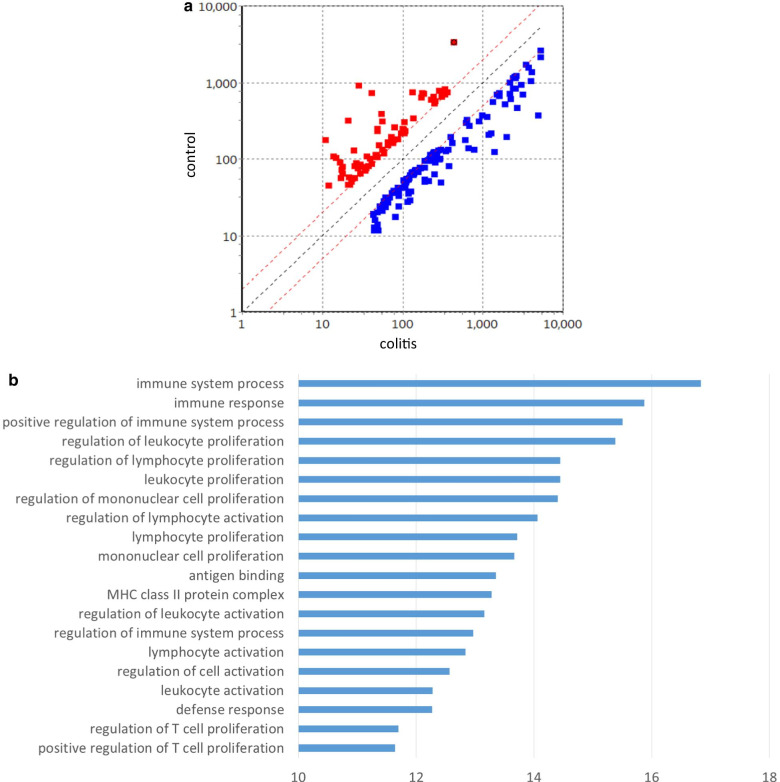
Microarray analysis of differentially expressed genes in colon biopsy samples obtained from patient with CML. (**a**) There were 194 genes shown to be differentially dysregulated (> 2- or < 0.5-fold difference, *p* < 0.05) in inflamed as compared with normal mucosa, as shown in the scatter plot. Up- and down-regulated genes are represented in blue and red, respectively. (**b**) Gene ontology analysis of transcriptome in inflamed colonic mucosa of patient with dasatinib-induced colitis. The X-axis represents the -log_10_ (*p* value) of the given transcripts and the Y-axis shows a detailed description of the roles for the category

Of 53,617 transcripts represented by the microarray, 194 were differentially over- or under-expressed (> 2- or < 0.5-fold difference, adjusted p-value < 0.05) in inflamed colonic mucosa samples obtained at the diagnostic colonoscopy examination as compared to normal samples obtained at the follow-up colonoscopy examination. Of those 194 transcripts, 126 were expressed more abundantly and 68 less abundantly in colonic mucosa of our patient with colitis (Fig. 3A). A list of the 194 dysregulated transcripts for dasatinib-induced colitis is shown in Supplementary Table 1.

Levels of expression of lymphocyte-associated genes and chemokines were substantially increased in inflamed colonic mucosa samples. Moreover, gene ontology (GO) analysis of the entire transcriptome was performed to elucidate the biological meaning of the differentially expressed genes in inflamed colonic mucosa. Of 3214 functional categories examined, the top 20 for differential gene expression are presented in Fig. 3B. The most prominent biological processes shown in GO analysis were immune system process (GO: 0,002,376), immune response (GO: 0,006,955), positive regulation of immune system process (GO: 0,002,684), regulation of leukocyte proliferation (GO: 0,070,663), and regulation of lymphocyte proliferation (GO: 0,050,670) (Fig. 3B). GO analysis of the transcriptome for dasatinib-induced colitis also revealed that significant categories for highly expressed genes seemed to have a strong correlation with response to the mucosal immune system occurring on leukocytes, including lymphocytes in colonic mucosa.

## Discussion and conclusions

Hemorrhagic colitis is a unique adverse event that can occur during dasatinib treatment in CML patients and generally less common, though can easily be treated by stopping the drug administration [13]. Most affected patients notice onset by development of GI symptoms, such as bloody stool or abdominal pain. On the other hand, the present patient had no abdominal symptoms throughout the follow-up period and was diagnosed with colitis based on positive results for fecal occult blood in a colonoscopy examination. Dasatinib was carefully continued in this case, because we assessed the colitis as a grade 1 adverse event, which is defined as asymptomatic, and because of the presence of only pathologic or radiographic findings, as noted in the Common Terminology Criteria for Adverse Events (CTCAE). Furthermore, deep remission from CML was maintained patient during the observation period.

According to safety analysis findings obtained in previous clinical studies of CML patients with dasatinib, the prevalence of colitis is relatively low (1–10%) as compared to other GI symptoms, such as nausea (20–30%), abdominal pain (25%), and diarrhea (up to 40%) [14]. However, a recent study performed in Japan reported that CML patients receiving dasatinib treatment had a higher rate (33%) of hemorrhagic colitis. In those patients with colitis as well as the present case, a positive fecal occult blood test was the first indication prior to diagnosis and no GI symptoms during dasatinib therapy were noted. These observations suggest that CML patients should undergo regular fecal blood testing during dasatinib therapy, even when symptoms are absent. In addition, a total colonoscopy examination should be performed for early detection of colitis when a fecal blood test is positive.

As noted, the availability of TKIs targeting the BCR-ABL1 gene sequence has dramatically changed the outcome of patients with CML. However, those inhibitors are not entirely specific for BCR-ABL1, because tyrosine kinases share the same homology to a great extent, thus lack of specificity could lead to inhibition of numerous tyrosine kinases beyond the expected effect [15]. These so-called off-target effects, such as plural effusion seen in patients receiving dasatinib, have been shown to be associated with both clinical efficacy and toxicity, which cannot be explained by inhibition of BCR-ABL1. Colitis development may also be an off-target effect of dasatinib treatment. In the present case, significant lymphocytosis was continuously observed in peripheral blood samples obtained up to 9 months after diagnosis of CML. Recent studies have reported that clonal lymphocytosis, including large granular lymphocytes, were frequently present in peripheral blood samples taken from CML patients during dasatinib therapy [16], though no such finding was obtained in the present case. Interestingly, this phenomenon has only been reported to be induced by dasatinib, not other TKIs, suggesting it to be a dasatinib-specific off-target effect associated with other adverse events, such as colitis and pleural effusion.

In the present as well as some previously reported cases, IHC staining in biopsy samples of the colon clearly revealed cytotoxic T cell (CD3 + CD8 +) infiltration of the lamina propria, though the underlying mechanism has not been investigated [7]. In order to elucidate the pathogenesis of colitis, we performed transcriptome analysis of colon biopsy specimens from our CML patient with active colitis and compared the identified genes using microarray analysis. Those results confirmed that the transcript signature for dasatinib-induced colitis is composed of dysregulated genes involved in lymphocyte-mediated inflammation, which may play a central role in the pathogenesis of the disease. In addition, that speculation was strongly supported by results of GO analysis of the transcriptome, which indicated that functional categories among highly expressed genes on lymphocytes that have infiltrated epithelium of the colon are associated with local response for regulating the mucosal immune system.

The present case report provides details of dasatinib-induced asymptomatic colitis in a CML patient who was followed for more than 3 years, during which dasatinib was safely continued, except for a short period of withdrawal due to pleural effusion, and in whom DMR to CML was maintained. In addition, colitis associated with dasatinib did not seem to be exacerbated in colonoscopy findings obtained at follow-up examinations. These findings suggest that CML patients with dasatinib-induced colitis may be eligible for careful observation without drug cessation when no GI symptoms are present. Maintenance of treatment-free remission (TFR), defined as discontinued TKI therapy with MMR (≤ 0.1% BCR-ABL1 transcripts according to the international scale) and no need for treatment resumption has recently emerged as a new goal for many patients with CML after achievement of DMR [17]. The duration of DMR during TKI treatment might be a critical factor to maintain TFR. Thus, when CML patients with asymptomatic colitis develop abdominal symptoms, careful follow-up examinations, along with reduction in the dose of dasatinib or discontinued administration may be necessary to maintain DMR.

In conclusion, the present gene expression profile results for dasatinib-induced colitis demonstrate that lymphocyte-associated genes and chemokines are dysregulated to a considerable degree in colon mucosa. Furthermore, mucosal lymphocyte immunity may play an important role in development of colitis associated with dasatinib administration.

## Data Availability

All data generated or analyzed during this study are included in this article.

## Abbreviations

TKI: Tyrosine kinase inhibitor;
CML: Chronic myeloid leukemia
DMR: Deep molecular response
PDGFR-β: Platelet-derived growth factor receptor-β
GI: Gastrointestinal
CML: Cytomegalovirus
IHC: Immunohistochemical
GO: Gene ontology
CTCAE: The Common Terminology Criteria for Adverse Events
TFR: Treatment free remission
MMR: Major molecular response
